# The challenge of pneumonia & acute diarrhoea at global, regional & national levels: Time to refocus on a top most priority health problem

**Published:** 2011-02

**Authors:** Alok Kumar Deb, Suman Kanungo, G. Balakrish Nair, Jai P. Narain

**Affiliations:** * National Institute of Cholera & Enteric Diseases (ICMR), Kolkata, India; + World Health Organization, Regional Office for South-East Asia, New Delhi, India

## The burden

In the developing countries, communicable diseases contribute to an enormous share of disease burden in all age groups, especially children^1^–^3^. Of 8·79 million children below the age of five who die worldwide each year, 68 per cent (5·97 million) die due to infectious diseases - the major contributors being pneumonia (18% or 1.7 million deaths) and diarrhoea (15% or 1.5 million deaths)^4^. Five countries: India, Nigeria, Democratic Republic of the Congo, Pakistan, and China contributed nearly 50 per cent of these deaths. Nearly, 2.4 million (27.4% of the global figure) child deaths occur in the South-East Asia (SEA) Region alone; 21 and 14 per cent were contributed by pneumonia and diarrhoea, respectively. Among countries of this region, India suffers quite heavily from the global burden; out of 1.83 million child deaths, 20.3 and 13 per cent were caused by these two diseases. In the context of the aim of UN Millennium Development Goal 4 (MDG 4)^5^ to reduce mortality of under five children by two-thirds between 1990 and 2015, many countries in the SEA Region seem not to be on track to meet this target^6^. The magnitude of the problem is even more challenging when morbidities are considered. Significantly, the median incidence of diarrhoeal diseases in under five children in developing countries has not changed much since the early 1990s – it was 3.5 episodes per child-year in 1993^7^ vs. 3.2 episodes per child-year in 2003^8^.

Acute diarrhoea and pneumonias also present as public health emergencies and occur in outbreaks, affecting adults and children alike. The outbreaks of cholera affecting large geographic areas have recently been reported from India and Nepal^9^,^10^. Similarly, the emergence of a new influenza virus- pandemic (H1N1) 2009 last year spread from two countries in April to all the continents within a few months and influenza-associated deaths were attributed to pneumonia, especially among pregnant women and other high risk populations^11^. This and the SARS outbreak of 2003^12^ are some of the examples of acute diarrhoea and pneumonia outbreaks that continue to challenge public health as never before.

Since these diseases are also associated with under- development, poverty, and a less-than-effective health system, these pose a huge challenge to child survival in the countries of the Region, in particular India^13^. Clearly, without effective response to pneumonia and acute diarrhoea, MDG4 will not be achieved^14^.

During the past several decades, worldwide research on these two leading killers of children and adults has generated huge and convincing evidence on easy, efficient, as well as cost-effective measures that can substantially reduce the burden of pneumonia and diarrhoea even in resource-poor settings^15^–^19^. These include promotion of early and exclusive breastfeeding and adequate nutrition, micronutrient supplementation, appropriate hygienic practices, safe water and adequate sanitation, appropriate home care and care-seeking practices, and complete and timely immunization, besides advocacy to policymakers regarding provision of additional and newer vaccines (such as Hib, Pneumococcal and rotavirus vaccine, and cholera vaccine where appropriate).

## Factors behind persistence of the problem

Despite the availability of the low cost and effective interventions- some developed in the Region, it is tragic to note the failure of health programmes to control these killer diseases even in the mid-21^st^ century. The possible reasons may be grouped into three levels –

- individual, household and community (low level of awareness, poverty, erroneous and often hazardous beliefs and norms, inadequate hygiene and sanitation),
- local government (deficient health infrastructure, inadequate provision of safe water and sanitation system, low health care expenditure, disproportionate funds allocation to different programmes, inadequate co-ordination and collaboration among different sectors and agencies), and
- international community (shortage of funds, ascribing inappropriate importance to other diseases or conditions, not pursuant with the needs of the country, lack of advocacy).

The key factors undoubtedly lie at the first level. However, at international level, the focus on these two diseases got lost over the past decade and half, in terms of both visibility as well as policy attention. As a result, in many countries the health-related resources and expenditure did not expand to match the increasing demands of scaling up of comprehensive set of proven effective interventions with gradual addition of extra components^20^. For example, apart from Timor Leste and the Maldives, other countries in the region did not have a significant rise in health expenditure (as percentage of GDP spent) during 1995-2006; some countries even had a decrease in spending^21^.

This disturbing scenario was further aggravated by two other factors – (*i*) recent global economic recession that caused a cut-down in health care and health research spending, and (*ii*) inappropriate shifting of focus to other diseases, based on demands by the external funding agencies and overlooking the real needs of the countries^22^,^23^. For example, there are large global funding windows for the diseases targeted for HIV, TB and Malaria but fewer windows exist for diarrhoea and pneumonia prevention and control each of which causes more deaths than HIV, TB and malaria together.

## Investing in pneumonia and diarrhoea: The next steps

For a significant reduction in morbidity and mortality in the developing countries for pneumonia and diarrhoea, evidence-based interventions are required. A comprehensive package of prevention and case management intervention, implemented and scaled up using an integrated and inter-sectoral approach is needed. The package would include not only treatment of diarrhoea with ORS or pneumonia with antibiotics, but also addressing risk factors such as nutrition, breast-feeding, environmental aspects as well as underlying societal factors such as inequity and lack of access by poor and socially vulnerable. The combination of interventions to be scaled up would vary from country-to-country, depending on prevailing situations. Although regional and global collaboration is critical, the effectiveness of future policies to deal with the burden of communicable diseases in the region will only be assured if these policies are based on proper evidence. Thus, it will be very crucial to generate country-specific evidence on an urgent basis on factors that hinder the implementation of the well documented and effective control strategies and the ways to overcome such hindrances. This could logically be achieved by initially conducting a quick yet robust evaluation of different aspects of the existing control programme, followed by taking appropriate measures based upon the evidence generated thereby.
